# Predicting response to immunotherapy in lung cancer: an early HTA of predictive tests

**DOI:** 10.1017/S0266462325100317

**Published:** 2025-07-07

**Authors:** Tim Govers, Evelien van Well, Rik De Wijn, Michel van den Heuvel

**Affiliations:** 1 Department of Medical Imaging, Radboud University Medical Center, Nijmegen, The Netherlands; 2 Medip Analytics, Nijmegen, The Netherlands; 3 Pamgene International, Den Bosch, The Netherlands; 4 Department of Pulmonary Diseases, Radboud University Medical Center, Nijmegen, The Netherlands

**Keywords:** early HTA, cost-effectiveness, predictive biomarkers, lung cancer, QALY, costs

## Abstract

**Objectives:**

Predictive biomarkers can identify patients who are more likely to respond to immunotherapy, which can guide treatment decisions. The objective of this study was to assess the potential value of predictive biomarkers in advanced NSCLC patients to guide the development of cost-effective biomarkers in this field.

**Methods:**

A decision analytical model was constructed to compare theoretical new strategies with biomarkers to the current standard of care. The analysis was performed for three different patient groups based on PD-L1 status. Differences in health outcomes (QALYs) and costs were assessed between the current practice and these biomarker strategies.

**Results:**

Omitting immunotherapy in NSCLC patients with a PD-L1 score < 1 percent or between 1 and 49 percent, and a negative biomarker test, could potentially reduce healthcare costs significantly a small loss in QALYs. In these groups, a biomarker test is potentially cost-effective as the incremental cost-effectiveness ratio largely exceeds a willingness-to-accept threshold of €80,000 saved per QALY lost. For patients with a PD-L1 score > 50 percent, a considerable QALY gain can potentially be realized by adding chemotherapy to patients with a negative biomarker test. However, this comes at a significant increase in costs and appears not to be cost-effective.

**Conclusions:**

In general, predictive biomarkers seem to have the potential to increase the cost-effectiveness of treatment with immunotherapy in patients with advanced NSCLC. Optimal positioning of a biomarker depends on the weighing between health impact and costs.

## Introduction

Immune checkpoint inhibitors and targeted therapies have redefined treatment options for patients with advanced stage nonsmall cell lung cancer (NSCLC). Targeted therapies are only effective in tumors if specific genetic alterations they target are present, and the absence of these genetic alterations is a reality for most advanced NSCLC patients. For these patients, immune checkpoint inhibitors (i.e., immunotherapy) targeting either programmed cell death protein 1 (PD-1) or programmed cell death ligand 1 (PD-L1) have become important treatment options shown to be able to improve survival (1). However, long-term disease control with immunotherapy is only seen in a relatively small proportion of patients (2), whereas immunotherapy comes at the expense of side effects and significant monetary expenses. Therefore, treatment guidance based on response prediction is potentially valuable for these patients and society.

Predictive biomarkers can be used to guide treatment decisions by identifying patients who are more likely to respond to immunotherapy. Presently, only tumor PD-L1 expression is routinely used for this purpose in clinical practice. Multiple studies have shown that tumor PD-L1 expression is a predictive factor for response to immunotherapy (3–5). Although PD-L1 expression has been shown to be predictive for treatment response in advanced NSCLC, there is still a considerable proportion of patients with high PD-L1 expression who do not respond to immunotherapy. In contrast, other patients without PD-L1 expression still benefit from immunotherapy (6). Therefore, it is recognized that the development of additional biomarkers or combinations of biomarkers may be valuable to guide treatment decisions (7). Additional tests based on tumor tissue, such as Tumor Mutational Burden (TMB) and specific genomic alterations, are associated with the efficacy of immunotherapy and could be an addition to PD-L1 expression (8). Also, predictive models, based on clinical data or blood tests, have been proposed. The IOpener test is based on tyrosine kinase activity of immune cells in peripheral blood and provides a predictive score for the likelihood of response to immunotherapy (9).

To guide further research and development, it would be beneficial to first evaluate the potential value of these biomarkers using early health technology assessment (HTA). Early HTA is a methodology to assess the potential value of new healthcare technologies. Early HTA offers valuable insights about technology, like new biomarkers for predicting treatment effect in advanced NSCLC. It can highlight the advances of the new technology, show where it can be best positioned within current care pathways, provide information about the minimal needed performance, and give direction to future clinical studies (10;11).

In this study, we performed an early HTA about biomarkers for the prediction of immunotherapy response in advanced NSCLC patients to show the potential value of new biomarkers and to guide the development of cost-effective biomarkers in this field.

## Methods

### Target population and comparison

The target population consisted of Stage 3 and 4 NSCLC patients (as defined in the 8th edition of lung cancer staging) who were not eligible for (curative) chemoradiation and had no targetable mutations.

To analyze the potential value of new biomarkers in predicting immunotherapy response, we constructed a decision analytical model to compare different new strategies, including the use of biomarkers for prediction of treatment response, to the current standard of care. The analyses were performed for three different groups within the target population based on PD-L1 status: <1 percent, 1–49 percent, and ≥ 50 percent expression. These three groups were chosen based on the data presented in the KEYNOTE studies. The KEYNOTE studies are a series of clinical trials evaluating the effectiveness of Pembrolizumab, of which some are performed in NSCLC patients (12–14).

### Current standard of care

Standard of care was based on the Dutch guideline for the treatment of NSCLC and the European Society for Medical Oncology (ESMO) guideline (15;16). The current treatment for patients in the target population depends on the PD-L1 status:

- PD-L1 expression <1 percent or 1–49 percent: immunochemotherapy
- PD-L1 > 50 percent: immunotherapy alone.

Pembrolizumab was used as immunotherapy, and carboplatin in combination with pemetrexed or paclitaxel as chemotherapy in this study.

### Strategies including biomarkers

Theoretical strategies were defined in which a biomarker test was used to predict response to immunotherapy, and treatment was based on this prediction. Two possible outcomes for the biomarker test were considered: a positive test indicates that a response to immunotherapy is expected, whereas a negative test indicates that no response is expected to immunotherapy. Also, a binary definition for patient response was adopted for this modeling study: response was defined as progression-free survival (PFS) at 12 months.

In the theoretical strategies for patients with PD-L1 expression <1 percent, and 1 percent–49 percent, a positive biomarker test resulted in the use of immunochemotherapy (comparable to current standard of care), whereas a negative test resulted in the use of chemotherapy only, as no additive benefit of immunotherapy is expected for these patients based on the test results. For patients with PD-L1 expression of ≥50 percent, a positive result resulted in the use of immunotherapy alone, whereas patients with a negative biomarker test received immunochemotherapy. The rationale behind this strategy is that we assumed that patients could not be excluded from anti-PD1 immunotherapy because they have high PD-L1 expression. However, because the biomarker test predicts that there is no response, chemotherapy is added to increase the probability of response.

### Model structure

**Figure 1.**
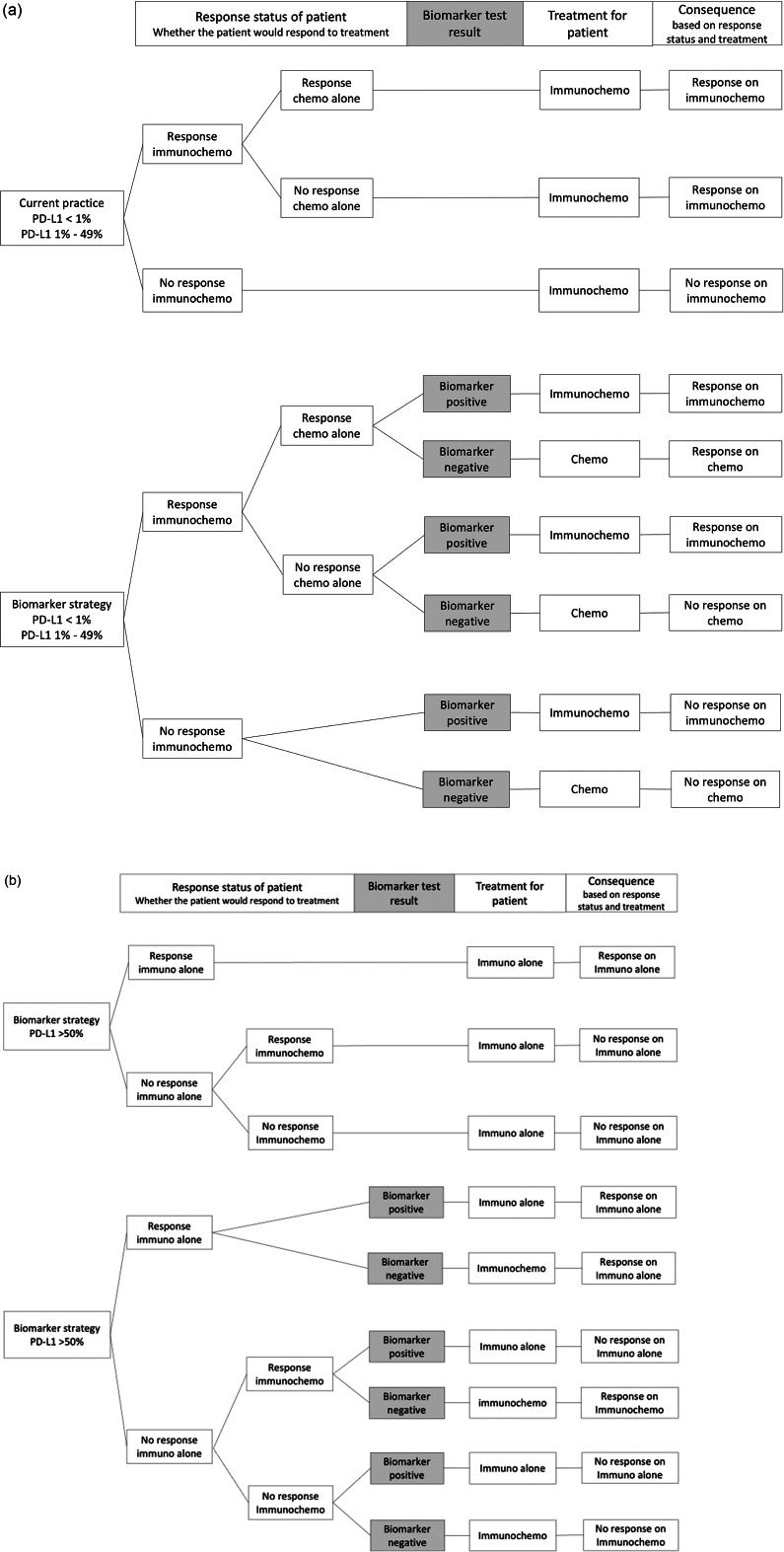
(A) Decision trees for current practice and the biomarker strategy for PD-L1 < 1% and PDL-1 1–49% groups. (B) Decision trees for current practice and the biomarker strategy for the PD-L1 ≥ 50% group.

**Figure 2.**
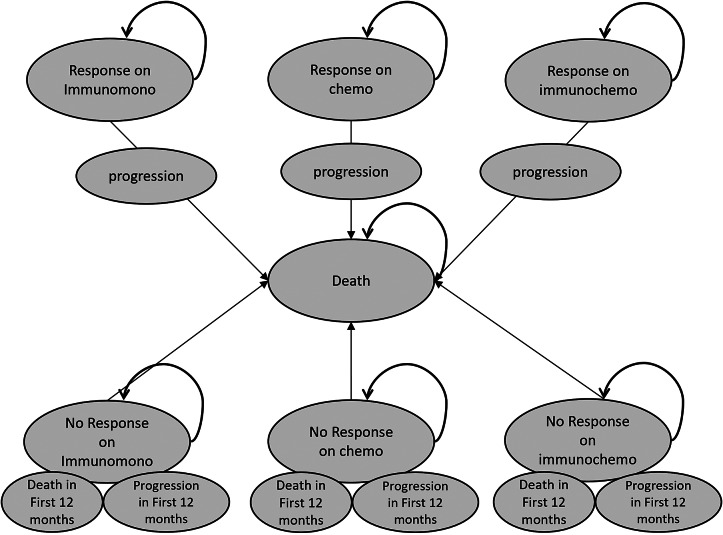
Markov model.

The model consists of a decision tree and a Markov model. The same patient cohort was simulated through both the current standard of care and the new theoretical strategies. In the decision tree, patients were first divided into groups associated with the response status, that is, whether they would respond if they received the therapy. In the current practice, all patients receive the same treatment (based on PD-L1 status), and the response status is used to define whether patients actually have a response (i.e., PFS) to treatment or not (consequence). Patients were divided between a negative or positive biomarker test in the biomarker strategy, resulting in different treatments. Based on the given treatment and the response status of the patient, the consequence regarding response was defined. The decision trees were the same for the <1 percent and 1–49 percent groups (Figure 1A). Figure 1B shows the decision tree for the group with PD-L1 expression >50 percent. Based on the decision tree, the patients were divided into different health states at the start of the Markov model. In the Markov model, the patients transition to another health state or stay in the same one. This happens in monthly cycles over a time horizon of five years. The same Markov model was applied for all three PD-L1 groups. Figure 2 shows the Markov model with the different health states. The model was developed in R. Lung cancer specialists were involved in the (clinical) validation of the model.

### Response

Whether or not patients will have a treatment response to a certain treatment (as included in the decision tree under the response status of the patient) was defined for each PD-L1 group based on different KEYNOTE studies. An overview of the response rates is given in Supplementary Material A.

For the PD-L1 < 1 percent group, response was based on a pooled analysis of KEYNOTE-021, KEYNOTE-189, and KEYNOTE-407 (12). This showed that 32.8 percent of patients respond to immunochemotherapy, whereas 19.3 percent respond to chemotherapy alone. It was assumed that patients with a response to immunochemotherapy may also show a response to chemotherapy alone, whereas patients who do not show a response to immunochemotherapy will also not respond to chemotherapy alone. This means that 58.7 percent (19.3 percent/32.8 percent) of the responders to immunochemotherapy would respond to chemo alone in the PD-L1 < 1 percent group. For the PD-L1 1 percent-49 percent response rates were based on KEYNOTE-189 (13). 42.9 percent of patients in this group respond to immunochemotherapy, whereas 17.7 percent respond to chemo alone. This means that 41.3 percent (17.7 percent/42.9 percent) of the responders to immunochemotherapy will respond to chemo alone.

For the PD-L1 > 50 percent group, no study has compared the response to immunotherapy alone and immunochemotherapy. Response to immunotherapy alone was obtained from KEYNOTE-042, which was 37.5 percent (17). To estimate the response to immunochemotherapy, we used data from the study of Li et al., who performed an indirect meta-analysis for this comparison (14). This indirect comparison showed an HR for progression-free survival of 1.81, resulting in a response rate of 65.5 percent for immunochemotherapy. We assumed that patients not responding to immunochemotherapy (34.5 percent) would also not respond to immunotherapy alone. Therefore, we calculated that 55.2 percent (34.5 percent/(1–37.5 percent) of the nonresponders to immunotherapy would not respond to immunochemotherapy. This means that 44.8 percent (100 percent-55.2 percent) of the nonresponders to immunotherapy would respond to immunochemotherapy.

### Nonresponders: progression and death in first 12 months

Nonresponders either died or had progression within the first 12 months after the start of therapy. The ratio between these two options was obtained from the difference in PFS and overall survival (OS) at 12 months in the KEYNOTE studies. The mortality rate for patients who died in the first 12 months was based on the presented OS curves of the different KEYNOTE studies. Details are described in Supplementary Material B.

For patients with progression within 12 months, it was assumed that progression occurred after 9 months on average, based on expert opinion. It was then assumed that they lived for an average of 5.4 months after the time of progression, which was based on a study about postprogression survival in advanced NSCLC patients (18).

### Disease progression after 12 months

Disease progression after the initial response was obtained from the PFS curves of the KEYNOTE studies for the different PD-L1 groups. Because it was not possible to differentiate between progression and mortality from these PFS curves, it was assumed that the first progression took place and patients would subsequently die from this disease progression. Therefore, patients moved from the response to treatment state to the progression states and finally to the death state at a rate inferred from the PFS curves. Then it was assumed that patients lived for an average of 5.4 months after progression, based on postprogression survival (18). Extrapolating the progression-free survival curves was done using an exponential function. Details are presented in Supplementary Material C.

In all Health states, patients had the probability of due to other causes. This mortality was based on the general mortality data from Statistics Netherlands (CBS).

### Utilities

Quality of life values in the form of utility values (with 0 for death and 1 for perfect health) were attached to the different health states. This allows for the calculation of quality-adjusted life years (QALYS) (19). These health outcomes were discounted at a rate of 1.5 percent based on the Dutch guideline for health economic evaluation (20).

A utility value for patients with response and patients with progression was assigned to the corresponding health states (21). Disutilities were extracted from these values attributed to the side effects of the treatment regimes (21–25). Disutilities of side effects were multiplied by the prevalence of these side effects and then aggregated as an average disutility in the health states. The prevalence of side effects was obtained from the KEYNOTE studies. An overview of the utilities and side effects for the different types of medication is presented in Supplementary Material A.

### Costs

Costs were based on costs in the Netherlands. Only healthcare costs were which consisted of biopsy costs, PD-L1 determination, medication costs, and costs for the treatment of side effects, were included. The cost of side effects was multiplied by the prevalence of these side effects and then aggregated as one cost in the health state. Furthermore, costs were assigned to patients who discontinued medication (one-time costs), the response state, progression state, and to patients who died (costs for terminal care). The cost of medication was assumed until the progression of the disease. Costs were indexed to a 2021 price level and discounted at a rate of 4.0 percent (20). An overview of the costs included in the model is presented in Supplementary Material A4. Details on the calculation of costs are given in Supplementary Material D.

### Analyses

To assess the potential value of biomarker tests that can predict the response to immunotherapy, several analyses were performed over a time horizon of five years. Differences in health outcomes (QALYs) and costs were assessed between the current practice and the strategies, including the biomarker test. Incremental cost-effectiveness ratios (ICER) were calculated by dividing the difference in costs by the difference in health outcomes. A willingness to pay (WTP) of €80,000 per QALY gained, or a willingness to accept (WTA) of €80,000 saved per QALY lost were used to decide whether the strategy including the biomarker was deemed cost-effective in a specific scenario (20). This WTP was used because of the high disease burden of NSCLC.

First, headroom analyses were performed in the different groups to show the maximum added value of a biomarker, that is, the value for a biomarker with perfect accuracy. This was done by setting sensitivity and specificity at 100 percent. For the PD-L1 < 1 percent and 1 percent-49 percent groups, this means that the test is always positive in patients who would respond to immunochemotherapy and always negative in patients who would not respond to immunochemotherapy. For the PD-L1 > 50 percent group, this means that the test is always positive in patients who would respond to immunotherapy alone, whereas the test is always negative in patients who would not respond to immunotherapy.

Next, three scenario analyses were performed regarding the sensitivity and specificity of the biomarker test, that is, scenario 1: 75 percent sensitivity and 75 percent specificity, scenario 2: 90 percent sensitivity and 60 percent specificity, and scenario 3: 60 percent sensitivity and 90 percent specificity. No costs for the biomarker test were included.

Sensitivity analyses were performed within the scenarios with 75 percent sensitivity and 75 percent specificity to show the influence of other parameters of the model.

## Results

### PD-L1 < 1 percent group

Treatment, according to current practice, resulted in 0.825 QALYs and €155,968 in costs per patient. Using the biomarker strategy with 100 percent sensitivity and 100 percent specificity (the headroom analysis), resulted in 0.780 QALYs and €125,654 in costs per patient. Hence, the biomarker strategy, in which immunochemotherapy was given based on the test result, resulted in a loss of 0.046 QALY at a cost saving of €30,314, corresponding to an ICER of €660,858 saved per QALY lost. This is deemed cost-effectivewith a WTA of €80,000 saved per QALY lost. The QALY loss was caused by higher survival rates of nonresponders on immunochemotherapy compared to nonresponders on chemotherapy within the first 12 months, that is, the death/progression ratio was more beneficial for nonresponders on immunochemotherapy. The other scenarios for biomarker sensitivity and specificity also resulted in a QALY loss combined with cost savings (Table 1), resulting in ICERS above the WTA threshold.

**Table 1.** Results for PD-L1 < 1% group

Analyses	QALY difference	Cost difference	ICER
Headroom	−0.046	-€30,314	€660,858 saved per QALY lost
Sens 75%/ spec 75%	−0.081	-€44,497	€549,497 saved per QALY lost
Sens 90%/ spec 60%	−0.046	-€26,886	€582,709 saved per QALY lost
Sens 60%/ spec 90%	−0.116	-€62,027	€536,258 saved per QALY lost

### PD-L1 1 percent-49 percent group

Current practice resulted in 0.903 QALYs and €175,837 in costs per patient. The headroom analysis resulted in 0.884 QALYs and €151,734 in costs for the strategy with the biomarker test. There with the biomarker strategy resulted in a reduction of 0.019 QALY at a cost saving of €24,103. This translates to an incremental cost-effectiveness ratio of €1,302,190 saved per QALY lost. This is deemed cost-effective with a WTA of €80,000 saved per QALY lost. All other scenarios also resulted in a QALY loss combined with cost savings (Table 2), resulting in ICERs above the WTA threshold.

**Table 2.** Results for PD-L1 1–49% group

Analyses	QALY difference	Cost difference	ICER
Headroom	−0.019	-€24,103	€1,302,190 saved per QALY lost
Sens 75%/ spec 75%	−0.093	-€46,893	€506,507 saved per QALY lost
Sens 90%/ spec 60%	−0.043	-€25,988	€610,258 saved per QALY lost
Sens 60%/ spec 90%	−0.140	-€67,799	€475,518 saved per QALY lost

### PD-L1 ≥ 50 percent group

When all patients with tumor PD-L1 ≥ 50 percent receive immunotherapy alone, defined as current practice, the average QALY was 0.842 at an average cost of €106,922. The headroom analysis for the biomarker strategy in which chemotherapy was added for patients with a negative test result, resulted in 1.369 QALYs and €247,347 in costs. This means that the biomarker strategy resulted in an increase of 0.527 QALY at a cost increase of €140,426. This translates to an incremental cost-effectiveness ratio of €266,264 per QALY gained. This is not deemed cost-effective with a WTP of €80,000 per QALY gained. All other scenarios also resulted in a QALY gained at extra costs (Table 3), resulting in ICERs above the WTP threshold.

**Table 3.** Results for PD-L1 > 50% group

Analyses	QALY difference	Cost difference	ICER
Headroom	+0.527	+€140,426	€266,264 per QALY gained
Sens 75%/ spec 75%	+0.479	+€137,539	€287,394 per QALY gained
Sens 90%/ spec 60%	+0.350	+€97,143	€277,832 per QALY gained
Sens 60%/ spec 90%	+0.608	+€177,953	€292,897 per QALY gained

Sensitivity analysis was performed in all groups in the scenario with 75 percent sensitivity and 75 percent specificity. The impact of uncertain input values was assessed by varying these input values in deterministic sensitivity analyses. First, the assumption regarding the 5.4 months average survival after progression was assessed. Changing this input value over 50 percent–150 percent of its original value did not alter any conclusions. Utility values in the progression and response states were also varied. Varying this between 50 percent of its original value and 1 percent did not alter any conclusions. Varying medication duration (different from the baseline assumption which was medication until progression) could have an impact on the group with PD-L1 > 50 percent. When immunotherapy and immunochemotherapy have the same total therapy duration the biomarker strategy in the PD-L1 > 50 percent group 1 was cost-effective. Results are shown in Supplementary Material E.

## Discussion

Immunotherapy with immune checkpoint inhibitors (ICI) has become a standard treatment for advanced NSCLC patients, for whom, in many cases, little other treatment options are available. However, treatment is only effective in some patients and the high cost of treatment is of concern as healthcare budgets are limited. Therefore, it is important to assess strategies that can improve the cost-effectiveness of treatments in this patient group. In this study, we performed an early HTA analysis to assess if applyinga biomarker strategy to guide treatment selection is potentially cost-effective. Previously, multiple studies have assessed the cost-effectiveness of biomarker testing for targetable mutations in NSCLC (26). As far as we know, this is the first assessment of the potential value of biomarkers for immunotherapy guidance.

In the standard of care situation patients with a PD-L1 score < 1 percent and between 1–49 percent are treated with immunochemotherapy and the results of our modeling assessment indicate that providing immunotherapy to only those NSCLC patients who are likely to respond, based on a biomarker test, could potentially save a significant amount of healthcare costs against a small loss in QALYs. For these patients, a biomarker test is potentially cost-effective as the ICER largely exceeds a WTA of €80,000 saved per QALY lost. Important to note is that the model showed that omitting immunotherapy in patients with a PD-L1 score < 50 percent is cost-effective even when the biomarker test is of low accuracy and even at values of 0 percent sensitivity and 0 percent specificity. This is because immunotherapy, in the manner it is currently being utilized, and with the costs used in these analyses, does not seem to be cost-effective (27). However, the model showed that increased accuracy of the biomarker test results in improved cost-effectiveness (higher costs saved per QALY lost).

In addition to testing performance and cost, other factors are important for the implementation of a test in practice. For instance, the test should allow for the results to be available in a timely manner. It is particularly beneficial if the test can be conducted on samples or tissues that are readily available through minimally invasive methods. Therefore, a test based on peripheral blood would be preferable over a test requiring tumor tissue. Also, because the heterogeneity of tumor tissue may be of concern for, for example, the determination of PD-L1 expression (28).

For patients with a PD-L1 score ≥ 50 percent, a considerable QALY gain can potentially be realized by adding chemotherapy for those patients who are unlikely to respond to immunotherapy alone. However, this comes at a significant increase in costs. Using a biomarker to guide treatment in this way might help to improve outcomes in cases where immunotherapy alone will likely not be effective, but this appears not to be cost-effective. In the model, it was assumed that increased survival would result in a longer use of these combined therapeutics, which resulted in a lower cost-effectiveness ratio for the biomarker test. If increased survival on immunochemotherapy would not extend the duration of therapy, the biomarker strategy could become cost-effective, as was shown in the sensitivity analyses. Also, a reduction in costs of (especially) immunotherapy could result in a cost-effective scenario of a biomarker in the PD-L1 ≥ 50 percent group. Under these conditions, developing a biomarker for this group can still be of interest.

In a decision analytical model, and especially in early HTA, several assumptions have to be made, resulting in some limitations to the study. First, we had to choose a definition for response. We used 12 months of PFS as the definition, which was a subjective choice. However, we assessed shorter time horizons of PFS (e.g., 3 months of PFS), and did this not change the conclusions of the analyses. Second, we had to make assumptions regarding progression and mortality of responders after twelve months. We assumed that all patients who “lost PFS” (based on the reduction of PFS) after 12 months were progressive, which ignores death due to other causes during this period. As mortality due to advanced NSCLC is high, this probably did not have a major effect on the outcomes. Third, we also had to assume the survival of patients after progression (either within the first 12 months or after the first 12 months). Based on the available literature, we assumed that patients had an average survival of 5.4 months, which might be an underestimation considering the overall survival from other studies. Sensitivity analyses showed, however, that this assumption does not seem to impact overall conclusions of the model, as the conclusions did not change even with an average of 10 months of postprogression survival. Fourth, no study is available that directly compared the outcomes of patients with PD-L1 > 50 percent on immunotherapy and immunochemotherapy. We therefore had to use a hazard ratio from an indirect meta-analysis, which makes the outcome of this analysis more uncertain. Fourth, costs are based on Dutch healthcare costs, which may not necessarily apply to other countries. However, our model and inputs may be modified to explore the situation in different healthcare settings.

Immunochemotherapy is often the treatment of choice for patients with PD-L1 < 1 percent and 1–49 percent. Introduction of a biomarker strategy in which patients are not treated with immunotherapy based on a predictive test will most likely result in omitting immunotherapy for some patients who could have benefited. As a result, the modeling shows these strategies result in small losses in QALYs even at high sensitivity. From both the patient’s and physician’s standpoint, this may be problematic as even small probabilities of response might be hard to withhold from patients. On the other hand, there will likely exist a threshold at which physicians, and even patients, would withhold from therapy. Potentially this could be the case when considering shorter time intervals for the definition of response, for example, using 3 months PFS instead of 12 months. When implementing biomarker-guided treatment, this threshold (including the definition of response) must be further explored. On the other hand, it is important to discuss the potential cost-effectiveness of patient selection and the option of including strategies with high cost savings at the expense of a small reduction in the number of QALYs. Especially considering the rising costs of healthcare and the concerns of its affordability.

In addition to the strategies discussed above, other biomarker strategies may be envisaged that have a beneficial health impact as well as acceptable cost-effectiveness. For example, a positive predictive test may treat patients with low PD-L1 expression with immunotherapy instead of immunochemotherapy. Also, new (immuno)therapy options for advanced NSCLC patients will emerge, such as combined CTLA4 and PD1 checkpoint blockers, and predictive biomarkers can be useful in deciding which patients should receive these new alternatives and for which patients the current immunotherapy is likely to suffice. A predictive biomarker may also be used as an additional tool to help decide if a patient should receive immunotherapy in the context of other clinical and laboratory findings.

In conclusion, in this early HTA, some potential new strategies for using a biomarker test for response prediction were assessed. Other additional strategies are also possible, and this analysis could serve as an example for assessing these new biomarker strategies in the future. For example, a strategy in which a combination of immunotherapies is provided based on the results of a biomarker test could be explored. Furthermore, this study can be used as an example to guide optimal cut-off points for biomarker tests. This early HTA can be used to guide decisions in the research and development of tests that are able to predict the response to immunotherapy. In later stages of development of such tests, more extensive (sensitivity) analyses of cost-effectiveness should be performed to guide treatment decisions.

## Supporting information

Govers et al. supplementary material Govers et al. supplementary material

## Supplementary material

The supplementary material for this article can be found at http://doi.org/10.1017/S0266462325100317.
