# Supplementary material for: Predicting response to immunotherapy in lung cancer: an early HTA of predictive tests
Source: Int J Technol Assess Health Care. 2025 Jul 7;41(1):e57. doi: 10.1017/S0266462325100317 (PMC12390746; doi:10.1017/S0266462325100317)
Supplement: Govers et al. supplementary material [file S0266462325100317sup001.docx]

**Supplementary material**

Supplement A: overview of input values

Probabilities

| **Parameter** | **Value** | **Source** |
| --- | --- | --- |
| *Response rates* | | |
| PD-L1 <1%: Response Immunochemo | 32.8% | Borghaei et al. (1) |
| PD-L1 <1%: Response Chemo alone | 19.3%* | Borghaei et al. (1) |
| PD-L1 <1%: response on Chemo alone in the group with responders on Immunochemo* | 58.7% | Borghaei et al. (1) |
| PD-L1 1%-49%: Response Immunochemo | 42.9% | Gadgeet et al. (2) |
| PD-L1 1%-49%: Response Chemo alone | 17.7% | Gadgeet et al. (2) |
| PD-L1 1%-49%: response on Chemo alone in the group with responders on Immunochemo* | 41.3%* | Gadgeet et al. (2) |
| PD-L1 >50%: Response Immunomono | 37.5% | Mok et al. (3) |
| PD-L1 >50%: Response on Immunochemo | 65.5% | Mok et al. (3) |
| PD-L1 >50%: Response on Immunochemo in the group without response on immunomonotherapy* | 44.8%* | Mok et al. (3) |
|  |  |  |
| *Non-responders* | | |
| PD-L1 <1% - immunochemotherapy: Death / Progression ratio | 48.5% / 51.5% | Borghaei et al. (1) |
| PD-L1 <1% - chemotherapy: Death / Progression ratio | 64.6% / 35.4% | Borghaei et al. (1) |
| PD-L1 1%-49% - Immunochemotherapy: Death / progression ratio | 49.5% / 50.5% | Gadgeet et al. (2) |
| PD-L1 1%-49% - chemotherapy: Death / progression ratio | 60.8% / 39,2% | Gadgeet et al. (2) |
| PD-L1 >50% - immunomonotherapy: Death / Progression ratio | 59.1% / 40.9% | Mok et al. (3) |
| PD-L1 >50% -Immunochemotherapy: Death / Progression ratio | 49.3%/ 50.7% | Mok et al. (3) |
|  |  |  |
| Mortality rate of non-responders in first 12 months | Supplementary A |  |
| Progression rate of responders after first 12 months | Supplementary B |  |

Utility Values

| **Parameter** | **Value** | **Source** |
| --- | --- | --- |
| Utility response | 0.673 | Nafees et al. (4) |
| Utility Progression | 0.473 | Nafees et al. (4) |
|  |  |  |
| *Disutility side effects* |  |  |
| Nausea & vomiting | -0.048 | Nafees et al. (4) |
| Anemia | -0.073 | Westwood et al. (5) |
| Fatigue | -0.074 | Nafees et al. (4) |
| Constipation | -0.001 | NICE guideline(6) |
| Diarrhea | -0.047 | Nafees et al. (4) |
| Neutropenia | -0.090 | Nafees et al. (4) |
| Dyspnea | -0.050 | Doyle et al. (7) |
| Rash | -0.033 | Nafees et al. (4) |
| Thrombocytopenia | -0.020 | Handorf et al. (8) |

Prevalence of side effects

| **Side effects** | **Prevalence immunochemo** | **Prevalence Chemo** | **Prevalence immunomono** |
| --- | --- | --- | --- |
| Nausea & vomiting | 3.5% | 3.5% | 0.0% |
| Anemia | 16.3% | 15.3% | 1.9% |
| Fatigue | 5.7% | 2.5% | 1.4% |
| Constipation | 1.0% | 0.5% | 0.0% |
| Diarrhea | 5.2% | 3.0% | 3.9% |
| Neutropenia | 15.8% | 11.9% | 0.0% |
| Dyspnea | 3.7% | 5.4% | 0.0% |
| Rash | 1.7% | 1.5% | 0.0% |
| Thrombocytopenia | 7.9% | 6.9% | 0.0% |

Costs

| **Cost parameter** | **Value (per month)** | **Source** |
| --- | --- | --- |
| PD-L1 test | €110 | Dutch Healthcare Institute (9) |
| Biopsy | €239 | Dutch Healthcare Institute (9) |
| Immunotherapy (Pembroluzimab) | €8,549 | Dutch Healthcare Institute and ‘farmacotherapeutisch kompas’ (9, 10) |
| Chemotherapy (Carboplatin + Pemetrexed/paclitaxel) first three months | €3,740 | Dutch Healthcare Institute and ‘farmacotherapeutisch kompas’ (9, 10) |
| Chemotherapy (Pemetrexed/Paclitaxel) > 3 months | €3,654 | Dutch Healthcare Institute and ‘farmacotherapeutisch kompas’ (9, 10) |
| Immunochemotherapy | €12,288 | Dutch Healthcare Institute and ‘farmacotherapeutisch kompas’ (9, 10) |
| Immunotherapy discontinuation | €2,137 | Dutch Healthcare Institute (9) |
| Chemotherapy discontinuation | €3,882 | Dutch Healthcare Institute (9) |
| Immunochemotherapy discontinuation | €2,137 | Dutch Healthcare Institute (9) |
| Response State | €217 | Dutch Healthcare Institute (9) |
| Progression state | €451 | Dutch Healthcare Institute (9) |
| Terminal care costs | €1,243 | Dutch Healthcare Institute (9) |
| Nausea & vomiting | €704 | Dutch Healthcare Institute (9) |
| Anemia | €1,888 | Dutch Healthcare Institute (9) |
| Fatigue | €787 | Dutch Healthcare Institute (9) |
| Constipation | - |  |
| Diarrhea | €2,281 | Dutch Healthcare Institute (9) |
| Neutropenia | €1,358 | Dutch Healthcare Institute (9) |
| Dyspnea | - |  |
| Rash | 765 | Dutch Healthcare Institute (9) |
| Thrombocytopenia | 324 | Dutch Healthcare Institute (9) |

Weighed costs and disutilities of side effects (i.e. the probabilities of side effects multiplied the costs and disutility values

|  | Immunochemo | Chemo | Immunomono |
| --- | --- | --- | --- |
| Costs side effects | €776 | €599 | €76 |
| Disutility side effects | -0.037 | -0.029 | -0.004 |

Supplement B: Cumulative Mortality rate patient who died in the first 12 months

| month | PD-L1 < 1% immunochemo | PD-L1 < 1%  chemo | PD-L1 1%-49% immunochemo | PD-L1 1%-49% chemo | PD-L1 ≥ 50% immuno | PD-L1 ≥ 50% immunochemo |
| --- | --- | --- | --- | --- | --- | --- |
| 1 | 0,083 | 0,030 | 0,055 | 0,070 | 0,151 | 0,151 |
| 2 | 0,130 | 0,115 | 0,095 | 0,070 | 0,283 | 0,283 |
| 3 | 0,242 | 0,194 | 0,247 | 0,138 | 0,354 | 0,354 |
| 4 | 0,319 | 0,255 | 0,353 | 0,140 | 0,503 | 0,503 |
| 5 | 0,397 | 0,365 | 0,469 | 0,276 | 0,578 | 0,578 |
| 6 | 0,465 | 0,421 | 0,550 | 0,380 | 0,687 | 0,687 |
| 7 | 0,584 | 0,543 | 0,581 | 0,551 | 0,743 | 0,743 |
| 8 | 0,663 | 0,688 | 0,637 | 0,621 | 0,804 | 0,804 |
| 9 | 0,715 | 0,761 | 0,806 | 0,827 | 0,858 | 0,858 |
| 10 | 0,804 | 0,862 | 0,915 | 0,862 | 0,897 | 0,897 |
| 11 | 0,932 | 0,932 | 0,943 | 0,932 | 0,941 | 0,941 |
| 12 | 1,000 | 1,000 | 1,000 | 1,000 | 1,000 | 1,000 |

Supplementary C: Progression after 12 month of patients with response in the first 12 months

Progression free survival after 12 months was based on the data from the KEY-NOTE studies.(1, 2, 11). Extrapolation of the progression free survival after the time-horizon for which data was available was done using an exponential function.

|  | PD-L1 < 1% immunochemo | PD-L1 1%-49% immunochemo | PD-L1 1%-49% chemo | PD-L1 ≥ 50% immunochemo |
| --- | --- | --- | --- | --- |
| Based on actual data until month | 38 | 32 | 26 | 40 |
| Progression probability (per month) based on extrapolation | 0.069 | 0.074 | 0.128 | 0.016 |

Supplement D: Details of cost calculations of medication costs

Costs of immunotherapy

|  | Immunotherapy (pembroluzimab) | Source |
| --- | --- | --- |
| Needed packages (including spillage) | 4 | Dutch Healhcare Institute(9) |
| Costs per package | 1,430 | ‘Farmacotheraputisch Kompas’ (10) |
| Costs per 3 weeks | 5,721 | - |
| Costs per month | 8,264 | - |
| Costs for application | 285 | Dutsch Healthcare Institute(9) |
| Total costs per month | 8,549 |  |

Costs of Chemotherapy

|  | Pemetrexed | Cisplatin (first three months) |
| --- | --- | --- |
| Dosage | 500 mg/m^2^ per 3 weeks | 75 mg/m^2^ |
| Average weight or body surface | 1.7 m^2^ | 1.7 |
| Total dose | 850 | 127.5 |
| Contents packaging | 500 mg | 10 mg |
| Needed packages (including spillage) | 2 | 13 |
| Costs per package | 1199 | 4,58 |
| Costs per 3 weeks | 2398 | 60 |
| Costs per month | 3464 | 86 |
| Costs for application | 190 | No extra costs |
| Total costs per month | 3,654 | 86 |

Chemotherapy could also exist of Paclitaxel and Carboplatin. Costs are comparable to Pemetrexed and Cisplatin.

Supplement E: Results of sensitivity analyses (in scenario with 75% sensitivity and 75% specificity)

PD-L1 < 1 %

| **Analyses** | **QALY difference** | **Cost difference** | **ICER** |
| --- | --- | --- | --- |
| Base Case | -0.081 | -€44,497 | €549,497 saved per QALY lost |
| Post progression survival + 50% (8.1 months) | -0.087 | -€44,549 | €510,257 saved per QALY lost |
| Post progression survival - 50% (2.7 months) | -0.075 | -€44,409 | €595,439 saved per QALY lost |
| Same medication duration (average 12 months) | -0.083 | -€60,615 | €728,739 saved per QALY lost |

PD-L1 1%-49%

| **Analyses** | **QALY difference** | **Cost difference** | **ICER** |
| --- | --- | --- | --- |
| Base Case | -0.093 | -€46,893 | €506,507 saved per QALY lost |
| Post progression survival + 50% (8.1 months) | -0.099 | -€46,973 | €495,706 saved per QALY lost |
| Post progression survival - 50% (2.7 months) | -0.0853 | -€46,815 | €548,791 saved per QALY lost |
| Same medication duration (average 12 months) | -0.096 | -€55,409 | €576,728 saved per QALY lost |

PD-L1 ≥ 50 %

| **Analyses** | **QALY difference** | **Cost difference** | **ICER** |
| --- | --- | --- | --- |
| Base Case | +0.479 | +€137,539 | €287,394 per QALY gained |
| Post progression survival + 50% (8.1 months) | +0.478 | +€137,485 | €287,872 per QALY gained |
| Post progression survival - 50% (2.7 months) | +0.479 | +€137,553 | €286,917 per QALY gained |
| Same medication duration (average 12 months) | +0.501 | +€30,669 | €61,207 per QALY gained |

Additional information, including Markov Traces, can be requested from the corresponding author.

**References**

1. Borghaei H, Langer CJ, Paz-Ares L, Rodriguez-Abreu D, Halmos B, Garassino MC, et al. Pembrolizumab plus chemotherapy versus chemotherapy alone in patients with advanced non-small cell lung cancer without tumor PD-L1 expression: A pooled analysis of 3 randomized controlled trials. Cancer. 2020;126(22):4867-77.

2. Gadgeel S, Rodriguez-Abreu D, Speranza G, Esteban E, Felip E, Domine M, et al. Updated Analysis From KEYNOTE-189: Pembrolizumab or Placebo Plus Pemetrexed and Platinum for Previously Untreated Metastatic Nonsquamous Non-Small-Cell Lung Cancer. J Clin Oncol. 2020;38(14):1505-17.

3. Mok TSK, Wu YL, Kudaba I, Kowalski DM, Cho BC, Turna HZ, et al. Pembrolizumab versus chemotherapy for previously untreated, PD-L1-expressing, locally advanced or metastatic non-small-cell lung cancer (KEYNOTE-042): a randomised, open-label, controlled, phase 3 trial. Lancet. 2019;393(10183):1819-30.

4. Nafees B, Stafford M, Gavriel S, Bhalla S, Watkins J. Health state utilities for non small cell lung cancer. Health Qual Life Outcomes. 2008;6:84.

5. Westwood M, Joore M, Whiting P, van Asselt T, Ramaekers B, Armstrong N, et al. Epidermal growth factor receptor tyrosine kinase (EGFR-TK) mutation testing in adults with locally advanced or metastatic non-small cell lung cancer: a systematic review and cost-effectiveness analysis. Health Technol Assess. 2014;18(32):1-166.

6. Chronic obstructive disease in over 16s: diagnosis and management: NICE Guideline.

7. Doyle S, Lloyd A, Walker M. Health state utility scores in advanced non-small cell lung cancer. Lung Cancer. 2008;62(3):374-80.

8. Handorf EA, McElligott S, Vachani A, Langer CJ, Bristol Demeter M, Armstrong K, Asch DA. Cost effectiveness of personalized therapy for first-line treatment of stage IV and recurrent incurable adenocarcinoma of the lung. J Oncol Pract. 2012;8(5):267-74.

9. Zorginstituut Nederland: Farmacotherapeutisch rapport pembrolizumab (Keytruda) bij de behandeling van niet-kleincellig longkanker met PD-L1-expressie. 2016.

10. Farmacotherapeutisch Kompas 2022 [Available from: <https://www.farmacotherapeutischkompas.nl/>.

11. Li L, Xu F, Chen Y, Ren X, Liu Y, Chen Y, Xia S. Indirect comparison between immunotherapy alone and immunotherapy plus chemotherapy as first-line treatment for advanced non-small cell lung cancer: a systematic review. BMJ Open. 2020;10(11):e034010.
